# Intravital Visualization of Tattoo Particles After Picosecond Laser Treatment

**DOI:** 10.1111/srt.70283

**Published:** 2025-10-25

**Authors:** Lynhda Nguyen, Christian Mess, Volker Huck, Stefan W. Schneider, Katharina Herberger

**Affiliations:** ^1^ Laser Department, Department of Dermatology and Venereology University Medical Center Hamburg‐Eppendorf Hamburg Germany; ^2^ Department of Dermatology and Venereology University Medical Center Hamburg‐Eppendorf Hamburg Germany

**Keywords:** intravital imaging, multiphoton tomography, picosecond laser, tattoo

## Abstract

**Background:**

Picosecond (ps) laser treatments have been widely used for several years to remove unwanted tattoos. It is hypothesized that following the laser‐induced fragmentation of tattoo particles, transepidermal clearance occurs as one of the elimination processes alongside with the renewal of the skin. Nevertheless, the precise microscopic details of the tattoo clearance process following laser treatment remain unclear.

**Objective:**

To analyze the transepidermal clearance of tattoo particles, along with the morphological and metabolic changes in the surrounding tissue, following ps laser treatment.

**Material and Methods:**

The study population comprised healthy male and female patients seeking laser‐assisted tattoo removal, who were recruited from the Laser Department at the University Medical Center Hamburg–Eppendorf. Each subject underwent a single ps laser treatment session, and follow‐up assessments were conducted at 6 and 12 weeks post‐treatment using multiphoton tomography with fluorescence lifetime imaging (MPT‐FLIM).

**Results:**

The study included a total of nine participants with eleven tattoos. In untreated skin, the tattoo particles were observed to be confined to the dermis, situated between collagen bundles. Six weeks following treatment, tattoo particles were observed in both inter‐ and intracellular spaces across all epidermal and the upper dermal layers. By the 12‐week follow‐up, particles were still present in the epidermis and dermis, although their quantity appeared to have decreased. In accordance with the aforementioned findings, the mean fluorescence lifetime measurements demonstrated a decrease across the follow‐up visits, although they remained elevated at 12 weeks.

**Conclusion:**

Our in vivo, non‐invasive imaging data indicate that the transepidermal clearance of tattoo particles following ps laser treatment can extend over several months. This supports the hypothesis that longer intervals between ps laser treatments may be beneficial. Further prospective clinical studies are required to compare the efficacy and safety of short‐ and long‐term treatment intervals in laser‐assisted tattoo removal.

**Trial Registration:**

ClinicalTrials.gov identifier: NCT06431464

## 1 Introduction

Tattoos are a way of preserving memories, expressing oneself, or enhancing one's appearance. A permanent design is created by inserting pigments or inks into the dermis through punctures. Studies in 2019 found that prevalence of tattoos was approximately 28% in the USA, 37% in Germany, 17% in France, and 22% in Brazil, with these numbers steadily increasing [1, 2, 3, 4]. As tattoos become more popular, so does the demand for tattoo removal.

Laser tattoo removal is based on the principle of selective photothermolysis, in which laser energy is selectively absorbed by tattoo pigments that act as targeted chromophores [5]. Important parameters include pulse duration and thermal relaxation time (TRT) [6]. TRT is defined as the time it takes for a chromophore to lose 50% of the absorbed laser energy [7]. To minimize collateral damage, it is believed that the pulse duration must be less than the TRT of tattoo particles [6, 8].

Computer simulations suggested pulse duration in the picosecond (ps) range as a more effective treatment option [9]. In 2012, the FDA approved the use of ps lasers specifically designed to optimize tattoo removal [10]. Since then, ps lasers have established as an essential treatment modality in clinical practice, with numerous studies reporting on their efficacy and safety [11, 12, 13]. However, research into the removal process and the changes in surrounding tissue following ps laser treatment remains limited.

High‐resolution, non‐invasive imaging techniques are needed to effectively study the removal of tattoo particles after laser treatment. Although conventional histology and electron microscopy are widely used in both clinical and research settings, their ex vivo nature has limitations. Multiphoton tomography with fluorescence lifetime imaging (MPT‐FLIM) offers a promising alternative, providing subcellular resolution without the need for pretreatment. Previous studies have successfully visualized tattoo particles in human skin using MPT‐FLIM [14].

To the best of our knowledge, no studies have analyzed tattoo removal at the microscopic level after ps laser treatment. Therefore, this study aims to investigate the removal process, as well as the morphological and metabolic changes in the surrounding tissue, after ps laser treatment in living human skin.

## 2 Material and Methods

### 2.1 Study Design

This prospective, comparative study was approved by the ethics committee (2022‐100773‐BO‐ff), preregistered at clinicaltrials.gov, and conducted in accordance with the Declaration of Helsinki. The study population comprised healthy male and female volunteers with untreated black tattoos who were seeking laser treatment. These individuals were recruited from the Dermatological Laser Department at the University Medical Center Hamburg–Eppendorf. Individuals were excluded from participation if they were pregnant or breastfeeding, had lesions in the region to be treated, had been exposed to UV radiation within the previous 4 weeks, or had Fitzpatrick skin types IV–VI. Each participant underwent a single session of ps laser treatment, followed by a 6‐ and 12‐week follow‐up visit, which included MPT‐FLIM measurements.

### 2.2 Treatment Protocol

Prior to treatment, the region of interest was cleansed and disinfected with Octenisept solution (Schülke & Mayr GmbH, Norderstedt, Germany). Subsequently, a 1064 nm ps laser (PicoPlus, Lutronic Medical Systems, Hamburg, Germany) was applied, with supplementary air cooling (Cryo6; Zimmer Aesthetic Division) to minimize pain. The laser parameters (1064 nm wavelength, 4 mm spot size, 0.7–1.0 J/cm^2^) were set to attain the objective of tattoo whitening, which is the recommended clinical endpoint. Participants were advised to avoid sun exposure for 4 weeks following treatment. No local anesthesia or pain medication was required.

### 2.3 Multiphoton Tomography and Fluorescence Lifetime Imaging

In vivo measurements were conducted utilizing a CE‐certified multiphoton tomographic system (MPTflex, JenLab GmbH, Jena, Germany). A titanium:sapphire tunable laser system (Mai Tai, Newport Spectra‐Physics, Santa Clara, CA, USA) with a pulse duration of 100 fs was employed to excite endogenous fluorophores and second harmonic generation (SHG) using near‐infrared laser light. MPT‐FLIM provides a combination of morphological and biochemical information about the tissue without requiring exogenous dyes. The technical setup comprised a Glan calcite polarizer, two galvanometric mirrors, a beam expander, and a collimator. The laser pulses were directed into a 40× oil immersion objective lens with a numerical aperture of 1.3 (Carl Zeiss Jena GmbH, Jena, Germany). Wavelength‐specific fluorescence lifetime values and SHG signals were obtained using a photomultiplier (PMT) and time‐correlated single‐photon counting (TCSPC).

The biogenic fluorophores and the tattoo particles themselves of the tattoo particles were excited at a wavelength of 760 nm. For each area of interest, two or three z‐stacks were recorded from the corneal layer to a depth of 150 µm, with 1.5 µm increments. The recorded images had a resolution of 100 µm by 100 µm. FLIM data were recorded with an SPC 830 time‐correlated single‐photon counting (TCSPC) imaging module (Becker & Hickl GmbH, Berlin, Germany) and subsequently using SPCImage 8 software (SPCM, Becker&Hickl GmbH, Berlin, Germany). The mean fluorescence lifetime (τ_m_), which reflects the combined fluorescence signal from all excited fluorophores, including tattoo particles and endogenous molecules like NADH, was determined to assess the inflammatory state of the skin [15]. A two‐component exponential decay model, with freely varying lifetimes (τ_1_ and τ_2_) and amplitudes (a_1_ and a_2_), was used to fit the FLIM data. The resulting fluorescence lifetime data were false color‐coded, ranging from 0 ps (red) to 2000 ps (blue).

### 2.4 Statistical Analysis

The statistical analysis was conducted using GraphPad Prism (version 9, Graphpad Software, Boston, USA). The descriptive data are presented as means and standard errors of the mean (SEM). Unless otherwise stated, an unpaired *t*‐test was used to compare mean differences between groups. Statistical significance was determined at *p* < 0.05.

## 3 Results

### 3.1 Basic Characteristics

A total of nine patients with 11 tattoos underwent treatment. The mean age of the participants was 29.9 ± 5.3 years, with 67% of them being female. During the imaging process, a total of 91 z‐stacks were taken.

### 3.2 Visualization of Tattoo Particles in Human Skin

**FIGURE 1:**
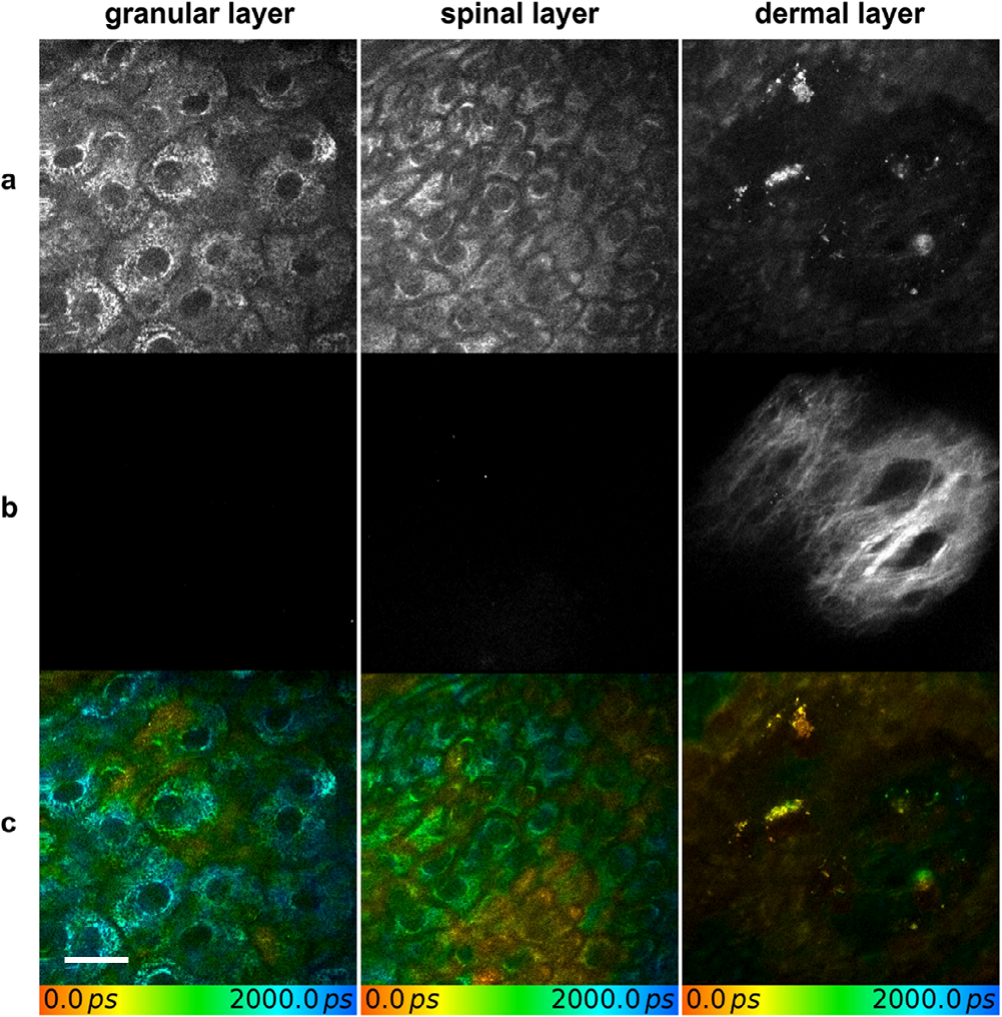
An old tattoo in human skin was visualized using multiphoton tomography combined with fluorescence lifetime imaging before picosecond laser treatment was employed. The granular, spinal, and basal/dermal layers were captured with the (**a**) multiphoton tomographic, (**b**) second harmonic generation, and (**c**) fluorescence lifetime imaging window. Old tattoo particles are observed in the dermis between collagen bundles, with no particles detected in the epidermis. Scale bar: 20 µm.

MPT‐FLIM was employed to visualize old tattoos in the dermal layer between collagen bundles before treatment. No tattoo particles were identified in the epidermal layers (**Figure** **1**). The cellular morphology was found to be similar to that of non‐tattooed skin. Tattoo particles were observed as well‐defined conglomerates of irregular shapes, with sizes varying from 1 µm to 11 µm. The fluorescence lifetime of the black tattoo particles was measured to be 105–125 ps, significantly shorter than that of most endogenous fluorophores, enabling their detection based on this characteristic short lifetime.

### 3.3 Tattoo Particles After Laser Treatment

**FIGURE 2:**
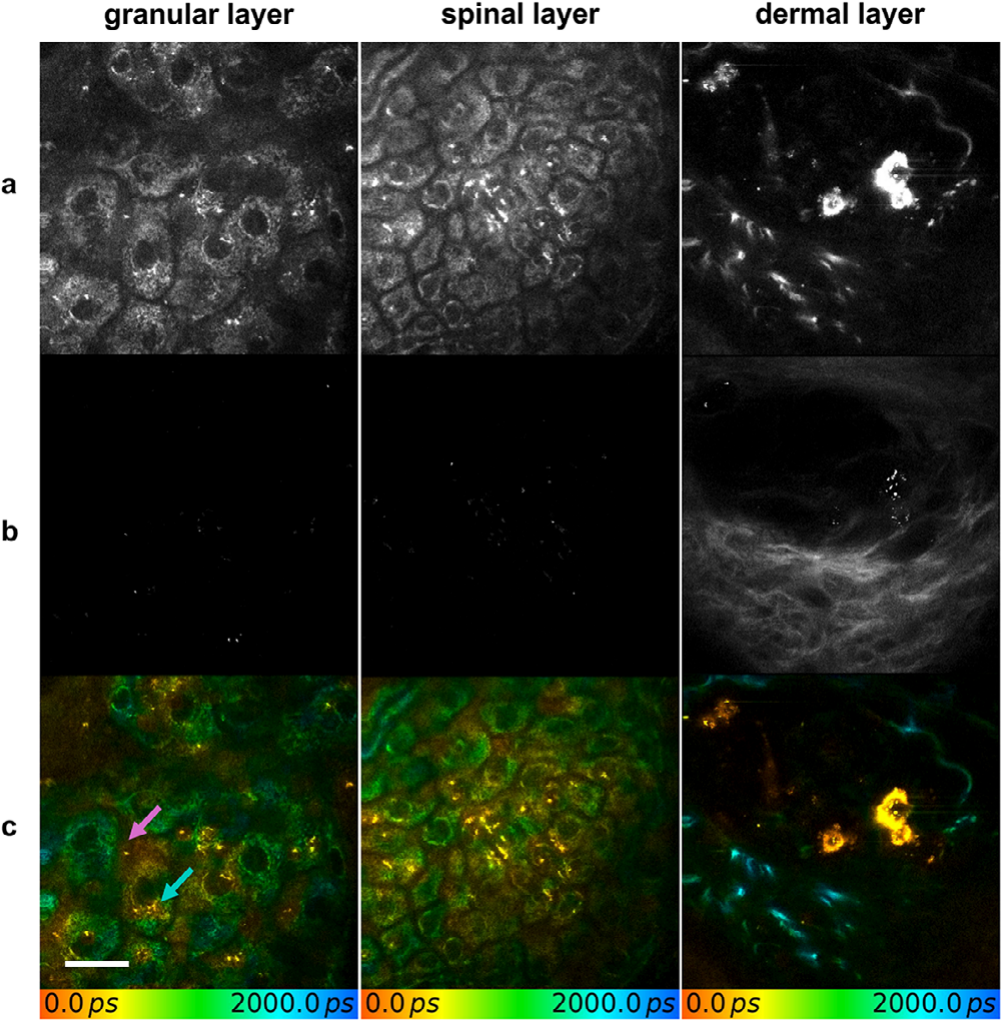
(a) Multiphoton tomographic, (b) second harmonic generation, and (c) fluorescence lifetime imaging of tattooed human skin 6 weeks after picosecond laser treatment. Tattoo particles of varying sizes were observed in both intercellular and intracellular spaces across all epidermal layers and the upper dermis, indicating ongoing transepidermal clearance. Purple arrow: intercellular localization. Blue arrow: intracellular localization. Scale bar: 20 µm.

Six weeks post‐treatment, it was found that the intercellular spaces had broadened and became irregular, indicative of edema associated with inflammation in the area treated with the laser. The presence of tattoo particles of varying sizes was observed in all layers of the epidermis as well as in the upper dermis. These particles were present in both intra‐ and intercellular spaces of keratinocytes (**Figure** **2**). In the dermis, the particles formed clusters around circular structures, indicating the potential for clearance via blood vessels.

**FIGURE 3:**
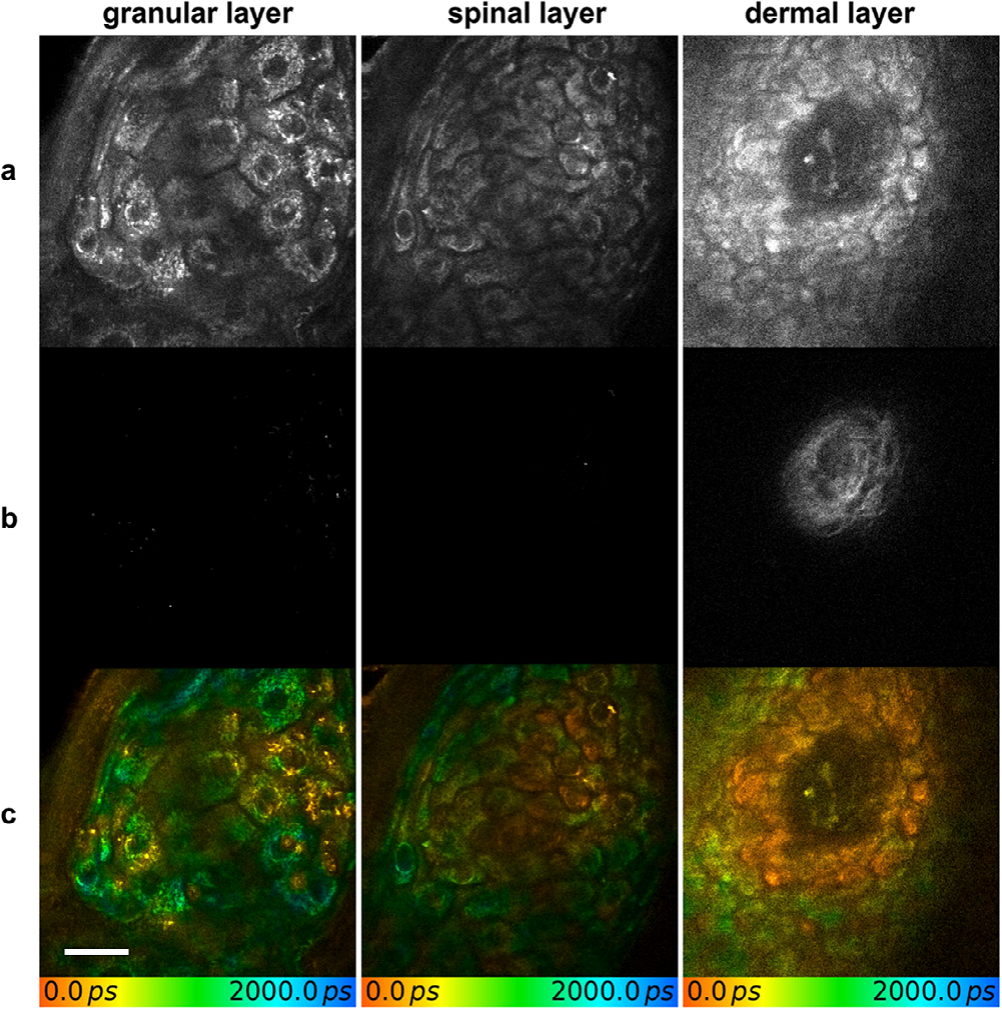
Using (a) multiphoton tomographic, (b) second harmonic generation, and (c) fluorescence lifetime imaging, tattoo particles could still be detected in all epidermal and the upper dermal layer 12 weeks post‐treatment. There seem to be fewer particles compared to the 6‐weeks follow‐up visit indicating a gradual clearance process even months after the last treatment session. Scale bar: 20 µm.

By 12 weeks post‐treatment, tattoo particles remained visible in both the epidermal and dermal layers, though their quantity had noticeably decreased compared to the 6‐week follow‐up, indicating a gradual clearance process (**Figure** **3**). The surrounding tissue showed no significant morphological changes compared to untreated controls. The fluorescence lifetime of the black tattoo pigments, measured at baseline and again at 6 and 12 weeks post‐treatment, remained unchanged, further confirming their identification.

### 3.4 Metabolic Alteration After Laser Treatment

The mean fluorescence lifetime (τ_m_) was calculated as an indicator of the cellular metabolic state. In untreated tattooed skin, the mean fluorescence lifetime (τm) value was found to be 1006.2 ± 178.5 ps. At 6 weeks post‐treatment, a significant increase in the mean fluorescence lifetime was observed, with τ_m_ rising to 1240.6 ± 146.5 ps (*p* < 0.05). This increase is likely to reflect a metabolic shift resulting from the inflammatory response following laser treatment. By 12 weeks post‐treatment, the mean fluorescence lifetime had decreased to 1119.8 ± 122.9 ps, although it remained elevated in comparison to the baseline (*p* < 0.05).

## 4 Discussion

Despite the long‐standing use of laser‐assisted tattoo removal, the timeline for transepidermal clearance of tattoo particles and the effects on surrounding tissue remain not fully understood. This study offers preliminary evidence of both transepidermal elimination and the long‐term morphological and metabolic effects on surrounding tissue following ps laser treatment for tattoo removal.

It is currently understood that laser‐assisted tattoo removal involves a number of complex mechanisms. It is postulated that due to the brief pulse duration, ps lasers are responsible for a predominantly photomechanical effect, which results in more efficient fragmentation of particles with minimal collateral damage, as opposed to a photothermal effect [16, 17, 18, 19]. Nevertheless, when targeting heterogeneous structures such as tattoo pigments, the potential for chemical alterations during the fragmentation process should be taken into account [20].

It is currently assumed that within approximately 4 weeks post‐treatment, the epidermis will be largely free of tattoo ink particles. This process is primarily driven by the natural renewal of the skin, whereby keratinocytes replace older cells, thereby facilitating the elimination of tattoo pigments [21, 22, 23]. Furthermore, the tattoo particles are phagocytosed by macrophages, which facilitate the removal of the tattoo particles through the transepidermal, lymphatic, and vascular system [24, 25]. However, the exact timeline for the complete removal of ink through these mechanisms has not been thoroughly investigated.

The findings of this study demonstrate a gradual transepidermal clearance of tattoo pigments, which continued even up to 12 weeks post‐treatment. These observations are consistent with the hypothesis that tattoo particles persist throughout all layers of the epidermis and the upper dermis, though their quantity visibly decreases over time. Correspondingly, mean fluorescence lifetime measurements demonstrated a decline across follow‐up visits, yet remained elevated at 12 weeks, indicating the persistence of inflammatory processes and pigment elimination weeks after the treatment.

To the best of our knowledge, the persistence of tattoo pigments in the epidermal layer 12 weeks post‐treatment remains not fully understood. This phenomenon is observed not only at the microscopic level but also clinically, as physicians frequently report continued lightening of tattoos even beyond the expected skin turnover period [26]. As a result, treatment intervals of at least 6 weeks or longer are generally recommended [27, 28]. One possible explanation for this prolonged clearance process would be the fragmentation of tattoo pigment conglomerates into numerous smaller particles. These particles may induce physical or chemical irritation, triggering a sustained inflammatory response in the skin. This ongoing inflammation could, in turn, extend the elimination process over a prolonged period, contributing to the continued lightening of the tattoo.

The results support the hypothesis that extending the interval between ps laser treatments to several months may be beneficial. Allowing for longer intervals may permit a greater opportunity for pigment clearance, which could potentially reduce the risk of complications and facilitate a more expedient recovery while simultaneously reducing the financial burden on patients. Nevertheless, further clinical studies are required to ascertain whether longer intervals between treatments offer superior or comparable outcomes to shorter intervals in terms of efficacy.

Furthermore, our study revealed no statistically significant alterations in the fluorescence lifetime of the black tattoo particles, indicating that their chemical composition remained not distinctly altered following the treatment. This finding supports the theory that ps lasers exert a primarily photomechanical effect, effectively fragmenting tattoo particles with minimal photothermal impact. Nevertheless, caution should be exercised when interpreting this result, as the complexity of tattoo particle composition and behavior under laser treatment may vary, necessitating further investigation to fully understand the morphological and chemical mechanisms at play in tattoo particles following laser treatment.

Emerging evidence suggests that fractional CO_2_ laser may enhance transepidermal pigment clearance by creating controlled microthermal zones, potentially improving elimination and skin texture [29, 30]. However, this approach has limitations, including potential prolonged post‐treatment downtime and a risk of post‐inflammatory hyperpigmentation. Despite these concerns, studies have shown promising outcomes. Au et al. reported reduced bulla formation with fractional CO_2_ and ps alexandrite laser [6], while Weiss and Geronemus observed enhanced clearance, faster recovery, and fewer side effects when combining fractional CO_2_ laser with a Q‐switched ruby laser [31, 32]. Additionally, biopsies in animal models have demonstrated pigment extrusion through the epidermis as necrotic debris following fractional laser treatment [33, 34]. Further prospective clinical and histological studies are needed to analyze clearance mechanism as well as efficacy and safety of this combination treatment approach.

It should be noted that this study is not without limitations. The study included only black tattoos, and as the participants received their tattoos from different studios, variations in ink formulations may have affected the results. Additionally, the deeper dermis could not be visualized due to the limited penetration depth of the MPT‐FLIM, which may have resulted in the exclusion of further information on the cutaneous clearance process. Moreover, the elimination process via the vascular and lymphatic systems, which are known to play a role in tattoo clearance, was not explored. Furthermore, like all microscopy techniques, MPT‐FLIM has a resolution limit. Although we observed a clear decrease in the number and size of detectable particles, we cannot rule out the presence of sub‐resolution particles. The autofluorescence of these unresolved particles directly contributes to the measured FLIM parameters, particularly influencing a_1_ and τ_1_. Another limitation is the missing photo documentation of the treated tattoos which would have enhanced the visual comparison of the efficacy of the laser treatment.

In conclusion, the in vivo, non‐invasive imaging data indicate that the process of transepidermal clearance of tattoo particles following ps laser treatments can extend over several months. This supports the hypothesis that longer intervals between ps laser treatments may be beneficial. Extending treatment intervals could facilitate more effective clearance of tattoo particles and potentially reduce the risk of complications, minimize recovery time, and improve overall patient outcomes. Additionally, no precise quantitative data regarding the rate of tattoo removal was provided. Consequently, removal may progress so slowly that shorter treatment intervals could be beneficial. However, clinical experience demonstrates that even with extended intervals between sessions, noticeable macroscopic lightening of the tattoo still occurs. Further studies are required to confirm these findings, particularly in relation to different tattoo colors and ink formulations, which may respond differently to laser treatment. Additionally, long‐term studies are necessary to fully understand the efficacy of extended treatment intervals and to enable comparison of the data with that obtained from nanosecond laser systems for achieving optimal tattoo removal outcomes.

## Conflicts of Interest

Lynhda Nguyen and Katharina Herberger have received lecture fees from Cynosure Lutronic. Christian Mess, Volker Huck, and Stefan W. Schneider have none to declare.

## Data Availability

The data that support the findings of this study are available from the corresponding author upon reasonable request.

## References

[1] A. E. Laumann and A. J. Derick , “Tattoos and Body Piercings in the United States: A National Data Set,” Journal of the American Academy of Dermatology 55, no. 3 (2006): 413–421. 16908345 10.1016/j.jaad.2006.03.026

[2] A. Borkenhagen , U. Mirastschijski , K. Petrowski , and E. Brahler , “[Tattoos in Germany: Prevalence, Demographics, and Health Orientation],” Bundesgesundheitsblatt, Gesundheitsforschung, Gesundheitsschutz 62, no. 9 (2019): 1077–1082. 31420716 10.1007/s00103-019-02999-7

[3] N. Kluger , L. Misery , S. Seite , and C. Taieb , “Tattooing: A National Survey in the General Population of France,” Journal of the American Academy of Dermatology 81, no. 2 (2019): 607–610. 30395921 10.1016/j.jaad.2018.10.059

[4] N. Kluger , S. Seite , and C. Taieb , “The Prevalence of Tattooing and Motivations in Five Major Countries Over the World,” Journal of the European Academy of Dermatology and Venereology 33, no. 12 (2019): e484–e486. 31310367 10.1111/jdv.15808

[5] R. R. Anderson and J. A. Parrish , “Selective Photothermolysis: Precise Microsurgery by Selective Absorption of Pulsed Radiation,” Science 220, no. 4596 (1983): 524–527. 6836297 10.1126/science.6836297

[6] L. Hernandez , N. Mohsin , F. S. Frech , I. Dreyfuss , A. Vander Does , and K. Nouri , “Laser Tattoo Removal: Laser Principles and an Updated Guide for Clinicians,” Lasers in Medical Science 37, no. 6 (2022): 2581–2587. 35604505 10.1007/s10103-022-03576-2

[7] L. I. Naga and T. S. Alster , “Laser Tattoo Removal: An Update,” American Journal of Clinical Dermatology 18, no. 1 (2017): 59–65. 27722955 10.1007/s40257-016-0227-z

[8] I. Kurniadi , F. Tabri , A. Madjid , A. I. Anwar , and W. Widita , “Laser Tattoo Removal: Fundamental Principles and Practical Approach,” Dermatologic Therapy 34, no. 1 (2021): e14418. 33068020 10.1111/dth.14418

[9] D. D. Ho , R. London , G. B. Zimmerman , and Y. DA , “Laser‐Tattoo Removal–A Study of the Mechanism and the Optimal Treatment Strategy via Computer Simulations,” Lasers in Surgery and Medicine 30, no. 5 (2002): 389–397. 12116333 10.1002/lsm.10065

[10] R. L. Torbeck , L. Schilling , H. Khorasani , J. S. Dover , K. A. Arndt , and N. Saedi , “Evolution of the Picosecond Laser: A Review of Literature,” Dermatologic Surgery 45, no. 2 (2019): 183–194. 30702447 10.1097/DSS.0000000000001697

[11] V. M. Hsu , A. S. Aldahan , S. Mlacker , V. V. Shah , and K. Nouri , “The Picosecond Laser for Tattoo Removal,” Lasers in Medical Science 31, no. 8 (2016): 1733–1737. 27056705 10.1007/s10103-016-1924-9

[12] Y. Qu , X. Feng , J. Liang , J. Liu , and D. Gao , “The Picosecond Laser Effects on Tattoo Removal and Metabolic Pathways,” Clinical Cosmetic and Investigational Dermatology 14 (2021): 1343–1350. 34594124 10.2147/CCID.S332265 PMC8478112

[13] O. Reiter , L. Atzmony , L. Akerman , et al., “Erratum To: Picosecond Lasers for Tattoo Removal: A Systematic Review,” Lasers in Medical Science 32, no. 2 (2017): 483. 27966053 10.1007/s10103-016-2130-5

[14] L. Nguyen , C. Mess , S. W. Schneider , V. Huck , and K. Herberger , “In Vivo Visualisation of Tattoo Particles Using Multiphoton Tomography and Fluorescence Lifetime Imaging,” Experimental Dermatology 31, no. 11 (2022): 1712–1719. 35837813 10.1111/exd.14646

[15] V. Huck , C. Gorzelanny , K. Thomas , et al., “From Morphology to Biochemical State—Intravital Multiphoton Fluorescence Lifetime Imaging of Inflamed Human Skin,” Scientific Reports 6 (2016): 22789. 27004454 10.1038/srep22789 PMC4804294

[16] D. D. M. Ho , R. London , G. B. Zimmerman , and Y. DA , “Laser‐Tattoo Removal—A Study of the Mechanism and the Optimal Treatment Strategy via Computer Simulations,” Lasers in Surgery and Medicine 30, no. 5 (2002): 389–397. 12116333 10.1002/lsm.10065

[17] V. M. Hsu , A. S. Aldahan , S. Mlacker , V. V. Shah , and K. Nouri , “The Picosecond Laser for Tattoo Removal,” Lasers in Medical Science 31 (2016): 1733–1737. 27056705 10.1007/s10103-016-1924-9

[18] O. Reiter , L. Atzmony , L. Akerman , et al., “Picosecond Lasers for Tattoo Removal: A Systematic Review,” Lasers in Medical Science 31 (2016): 1397–1405. 27311768 10.1007/s10103-016-2001-0

[19] W. Bäumler and K. Weiß , “Laser Assisted Tattoo Removal–state of the Art and New Developments,” Photochemical & Photobiological Sciences 18 (2019): 349–358. 30452057 10.1039/c8pp00416a

[20] P. Laux , T. Tralau , J. Tentschert , et al., “A Medical‐Toxicological View of Tattooing,” Lancet 387, no. 10016 (2016): 395–402. 26211826 10.1016/S0140-6736(15)60215-X

[21] M. Kuperman‐Beade , V. J. Levine , and R. Ashinoff , “Laser Removal of Tattoos,” American Journal of Clinical Dermatology 2 (2001): 21–25. 11702617 10.2165/00128071-200102010-00004

[22] M. M. Suter , K. Schulze , W. Bergman , M. Welle , P. Roosje , and E. J. Muller , “The Keratinocyte in Epidermal Renewal and Defence,” Veterinary Dermatology 20, no. 5‐6 (2009): 515–532. 20178490 10.1111/j.1365-3164.2009.00819.x

[23] P. J. Lea and A. Pawlowski , “Human Tattoo. Electron Microscopic Assessment of Epidermis, Epidermal‐Dermal Junction, and Dermis,” International Journal of Dermatology 26, no. 7 (1987): 453–458. 3654039 10.1111/j.1365-4362.1987.tb00590.x

[24] C. R. Taylor , R. R. Anderson , R. W. Gange , N. A. Michaud , and T. J. Flotte , “Light and Electron Microscopic Analysis of Tattoos Treated by Q‐Switched Ruby Laser,” Journal of Investigative Dermatology 97, no. 1 (1991): 131–136. 2056183 10.1111/1523-1747.ep12478570

[25] J. E. Ferguson , S. M. Andrew , C. J. Jones , and P. J. August , “The Q‐Switched Neodymium:YAG Laser and Tattoos: A Microscopic Analysis of Laser‐Tattoo Interactions,” British Journal of Dermatology 137, no. 3 (1997): 405–410. 9349338

[26] T. Kossida , D. Rigopoulos , A. Katsambas , and R. R. Anderson , “Optimal Tattoo Removal in a Single Laser Session Based on the Method of Repeated Exposures,” Journal of the American Academy of Dermatology 66, no. 2 (2012): 271–277. 22036610 10.1016/j.jaad.2011.07.024

[27] H. Alabdulrazzaq , J. A. Brauer , Y. S. Bae , and R. G. Geronemus , “Clearance of Yellow Tattoo Ink With a Novel 532‐nm Picosecond Laser,” Lasers in Surgery and Medicine 47, no. 4 (2015): 285–288. 25899971 10.1002/lsm.22354

[28] N. Saedi , A. Metelitsa , K. Petrell , K. A. Arndt , and J. S. Dover , “Treatment of Tattoos With a Picosecond Alexandrite Laser: A Prospective Trial,” Archives of Dermatology 148, no. 12 (2012): 1360–1363. 22986470 10.1001/archdermatol.2012.2894

[29] M. Radmanesh and Z. Rafiei , “Combination of CO2 and Q‐Switched Nd:YAG Lasers Is More Effective Than Q‐switched Nd:YAG Laser Alone for Eyebrow Tattoo Removal,” Journal of Cosmetic and Laser Therapy 17, no. 2 (2015): 65–68. 25411720 10.3109/14764172.2014.988724

[30] M. Vanarase , R. K. Gautam , P. Arora , S. Bajaj , N. Meena , and A. Khurana , “Comparison of Q‐Switched Nd:YAG Laser Alone Versus Its Combination With Ultrapulse CO(2) Laser for the Treatment of Black Tattoo,” Journal of Cosmetic and Laser Therapy 19, no. 5 (2017): 259–265. 28394665 10.1080/14764172.2017.1314506

[31] S. Au , A. M. Liolios , and M. P. Goldman , “Analysis of Incidence of Bulla Formation After Tattoo Treatment Using the Combination of the Picosecond Alexandrite Laser and Fractionated CO2 Ablation,” Dermatologic Surgery 41, no. 2 (2015): 242–245. 25590471 10.1097/DSS.0000000000000244

[32] E. T. Weiss and R. G. Geronemus , “Combining Fractional Resurfacing and Q‐Switched Ruby Laser for Tattoo Removal,” Dermatologic Surgery 37, no. 1 (2011): 97–99. 21073602 10.1111/j.1524-4725.2010.01821.x

[33] C. C. Wang , C. L. Huang , S. C. Lee , Y. M. Sue , and F. J. Leu , “Treatment of Cosmetic Tattoos With Nonablative Fractional Laser in an Animal Model: A Novel Method With Histopathologic Evidence,” Lasers in Surgery and Medicine 45, no. 2 (2013): 116–122. 23401095 10.1002/lsm.22086

[34] C. C. Wang , C. L. Huang , Y. M. Sue , S. C. Lee , and F. J. Leu , “Treatment of Cosmetic Tattoos Using Carbon Dioxide Ablative Fractional Resurfacing in an Animal Model: A Novel Method Confirmed Histopathologically,” Dermatologic Surgery 39, no. 4 (2013): 571–577. 23294007 10.1111/dsu.12104

